# The lost productivity cost of absenteeism due to COVID-19 in health care workers in Iran: a case study in the hospitals of Mashhad University of Medical Sciences

**DOI:** 10.1186/s12913-021-07200-x

**Published:** 2021-10-28

**Authors:** Ahmad Faramarzi, Javad Javan-Noughabi, Seyed Saeed Tabatabaee, Ali Asghar Najafpoor, Aziz Rezapour

**Affiliations:** 1 Department of Health Management and Economics, School of Public Health, Urmia University of Medical Sciences, Urmia, Iran; 2 Social Determinants of Health Research Center, Mashhad University of Medical Sciences, Mashhad, Iran; 3 Health Management Research Institute, Iran University of Medical Sciences, Tehran, Iran

**Keywords:** Health expenditures, Absenteeism, COVID-19, IRAN

## Abstract

**Background:**

Human resources management plays an important role in social development and economic growth. Absence from work due to health problems can make obstacles to the growth of economy. This study conducted aimed to estimate the absenteeism costs of COVID-19 among the personnel of hospitals affiliated to Mashhad University of Medical Sciences in Mashhad, Iran.

**Methods:**

This cross-sectional study was conducted between February 19, 2020, and September 21, 2020. The absenteeism costs were calculated using the human capital approach. Finally, we applied the linear regression to assess the impact of variables on the lost productivity of absenteeism due to COVID-19 among the personnel of hospitals affiliated to Mashhad University of Medical Sciences.

**Results:**

The results of this study showed that 1958 personnel had COVID-19. The total of absenteeism days in our study were 32,209 days, with an average of 16.44 absenteeism days. Total costs due to absenteeism were estimated to be nearly $1.3 million, with an average of $671.4 per patient. The results of regression model showed that gender (male), age (> 50 years), employment Type (non-permanent) and monthly income had a positive relationship with the absenteeism cost. Also, there are a negative significant relationship between absenteeism cost with job (physicians) and work experience.

**Conclusions:**

**A**bsenteeism costs of COVID-19 in the hospitals of Mashhad University of Medical Sciences represent a significant economic burden. The findings of our study emphasize the emergency strategies to prevent and control COVID-19 among the healthcare workers. It can decrease the economic impacts of COVID-19 and improve human resources management during the COVID-19 pandemic.

## Introduction

In December 2019, a few pneumonia cases with unknown etiology were first reported in Wuhan city, Hubei Province, China [1]. One week later, it was discovered that the pathogen of this pneumonia belonged to the coronavirus family, and the World Health Organization (WHO) officially named it COVID-19 on February 11, 2020 [2, 3]. The 2019 novel coronavirus disease (COVID-19) counts as the third outbreak caused by the coronavirus in the recent decades after Severe Acute Respiratory Syndrome (SARS) in 2002 and Middle East Respiratory Syndrome (MERS) in 2012 [4, 5]. SARS and MERS were not highly contagious compared to COVID-19. Overall, there were a total of more than 8000 confirmed cases of SARS in about 29 countries and more than 2000 cases of MERS across 27 countries [4, 6]. While until December 3, 2020, COVID-19 has involved approximately 220 countries and more than 64 million people and has caused the death of about 1.5 million people [7].

Iran was one of the first countries that experienced the outbreak of COVID-19, and the first confirmed cases of COVID-19 in Iran were reported on February 19, 2020 [8]. COVID-19 has rapidly spread throughout Iran and until December 03, 2020, have been reported over 1 million confirmed cases of COVID-19 and about 49,000 deaths [7]. Therefore, the COVID-19 pandemic has become a global health concern, which has generated an unprecedented impact on all aspects of life, including economic conditions [9]. One of the main adverse economic consequences of a contagious disease, such as COVID-19, can be decreased productivity due to the absence of sick staff from work [10]. In terms of health economics, the lost productivity costs due to absence from work (also called absenteeism costs) are a significant component of the health care costs. A study conducted in the United States (US) in 2010 reported that chronic obstructive pulmonary disease (COPD) was responsible for 16.4 million days absence of work. This study estimated that the absenteeism cost of patients with COPD was $3.9 billion [11]. Another study with 500 participants find that community-acquired pneumonia (CAP) causes 13 days of absenteeism [12].

It is reported that the COVID-19 pandemic is responsible for increases the risk of sickness absence in the general workforce, and particularly among healthcare workers [13, 14].

Healthcare workers play a key role in the treatment of patients with COVID-19 [15]. Healthcare workers are at higher risk for COVID-19 infection because they are at the forefront of the fight against the Covid 19 epidemic. As a result, they are more likely to quit their jobs and miss work [16]. Due to the importance of the role of health care personnel in controlling the Covid-19 epidemic, this study was conducted to estimate the cost of absenteeism due to COVID-19 among the personnel of hospitals affiliated to Mashhad University of Medical Sciences (MUMS). Mashhad, the center of Khorasan-e-Razavi province, is located in northeast of Iran. This city, with a population of about 3 million, is the second largest city in Iran and attracts more than 25 million pilgrims annually. In the Mashhad city, the MUMS is responsible for the health of the people of Mashhad. MUMS and its Subsidiary units consists of 25 hospitals with 22,000 personnel.

However, there is a lack of information regarding the days’ absence of work due to COVID-19 among the health care workers in Iran. Therefore, this study was designed to estimate the lost productivity cost of absenteeism due to COVID-19 among the personnel of hospitals affiliated to MUMS in Mashhad, Iran.

## Methods

### Study design and participants

We did a cross-sectional study to estimate the lost productivity cost of absenteeism due to COVID-19 among the personnel of hospitals affiliated to MUMS. The MUMS consists of 25 hospitals including 15 hospitals in Mashhad and 10 hospitals in the cities of Chenaran, Kashmar, Sarakhs, Roshtkhar, Dargaz, Khaf, Bardeskan, Taybad and Bakharz. The personnel of hospitals affiliated to MUMS are 22,000 people.

This study was conducted after receiving the Code of Ethics [IR.MUMS.REC.1400.001] from the Ethics Committee of the MUMS. In order to data collecting, the list of personnel who had taken sick leave due to COVID-19 between February 19, 2020, and September 21, 2020 was received by correspondence with all hospitals affiliated to MUMS. With the outbreak of Covid-19 and the importance of this disease, all hospitals affiliated to MUMS were required to follow up and register personnel with Covid-19 to prevent the spread of the virus and control the disease. All suspect and confirmed cases of COVID-19 who either had a positive PCR test or a letter from a physician stating that they had symptoms of COVID-19 could use corona sick leave. All corona sick leave was recorded in the health information system. Gender, age, residence, workplace, marital status, employment type, kind of job, work experience, monthly salary, and the missed workday number for every employee were extracted.

### Estimate absenteeism cost

The cost of productivity lost due to absence from work was estimated using the human capital approach. This approach is a method to calculate the indirect cost due to productivity loss. It is assumed the monetary value of losses productivity in the human capital approach, which can be caused by morbidity or premature death, is equivalent to the wage value in the absence of work [17]. Therefore, the period of absence from work due to illness is considered and valued by the achievable gross income.

We calculated the monetary value for a working day among employees who were absent due to COVID-19 and then multiplied by the number of missed workdays to estimate the absenteeism cost. The value monetary for a working day was computed using current salaries. All costs were converted into US dollars. The exchange rate used for conversion is 1 USD = 42,000 Iranian Rials.

### Statistical analysis

We first assessed the disease distribution among participants based on sociodemographic variables (gender, age, residence, workplace, marital status, employment type, kind of job, and work experience), we chose these variables because most studies have shown that sociodemographic variables influence COVID-19 distribution [18–20]. Then, we calculated the lost productivity cost of absenteeism due to COVID-19. The absenteeism costs are computed on an average and total. The mean, standard division, median, interquartile range (IQR), and percentage were used to describe costs according to variables. Besides, t-test and ANOVA were performed at a level significant of 5%. Finally, we applied the linear regression to assess the impact of variables on the lost productivity of absenteeism due to COVID-19 on health care workers. The regression model followed as below;

$$ {\mathrm{Y}}_{\mathrm{i}}=\upalpha +{\upbeta}_1{\mathrm{X}}_1+{\upbeta}_2{\mathrm{X}}_2+{\upbeta}_3{\mathrm{X}}_3+{\upbeta}_4{\mathrm{X}}_4+{\upbeta}_5{\mathrm{X}}_5+{\upbeta}_6{\mathrm{X}}_6+{\upbeta}_7{\mathrm{X}}_7+{\upbeta}_8{\mathrm{X}}_8+{\upbeta}_9{\mathrm{X}}_9+{\upbeta}_{10}{\mathrm{X}}_{10}+{\upvarepsilon}_{\mathrm{i}} $$

In this model, Y_i_ represents the dependent variable that is the total lost productivity cost of absenteeism for 7-month; X_1_ and X_2_ are gender and age, respectively; X_3_ and X_4_ represent the marital status and residence of participants; X_5_ to X_8_ are attributed to the patient’s job characteristics, including the type of employment and job, workplace, and experience; X_9_ is the natural logarithm of monthly income; X_10_ is the missed workday number; β is the vector of regression coefficients and shows the change in the total costs due to the change on independent variables; ε_i_ is the error term. The Shapiro–Wilk test and histogram were used to determine the normality of continuous variables. Continuous non-normal variables were logarithmically transformed.

All analyses were performed using Stata 14 (Stata Corp, College Station, Tex) software. Results were considered statistically significant if *p* < 0.05.

## Results

We identified 1958 cases with sick leave due to COVID-19 among the personnel of hospitals affiliated to MUMS (%8.9 of total personnel) during the study period, between February 19, 2020, and September 21, 2020. The majority of patients were female, 30–40 years of old, married and lived in Mashhad. Most of personnel were nurse, with 8 years or more of work experience, with non-permanent employment type. Socio-demographic characteristics of the study population are shown in Table 1.

**Table 1:** The Sociodemographic for the patient with COVID-19

Variable	Number of patients (percent)
**Gender**	
Male	781 (39.89)
Female	1177 (60.11)
**Age**	
< 30 years	468 (23.9)
30 to 40 years	970 (49.54)
41 to 50 years	415 (21.2)
> 50 years	105 (5.36)
Mean (years)	35.76
**City of residence**	
Mashhad	1531 (78.19)
Other cities	427 (21.81)
**Workplace**	
Small hospital (< 200 bed)	669 (34.17)
Large hospital (> 200 bed)	1118 (57.1)
Medical emergency	171 (8.73)
**Marital status**	
Married	1356 (69.25)
Single	393 (20.07)
Other	209 (10.67)
**Employment type**	
Permeant	867 (44.28)
Non-permanent	1091 (55.72)
**Job**	
Nurse	1077 (55.01)
Physician	47 (2.4)
Paramedic staff	252 (13.13)
Other staffs	577 (29.47)
**Work experience**	
**< =** 8 year	1007 (51.43)
> 8 year	799 (40.81)
Not available	152 (7.76)
Mean (years)	8.61
**Number of missed work day**	32,209
**Mean (days)**	16.44

Table 2 shows the lost productivity cost due to absenteeism among patients with COVID-19 by the sociodemographic variables. Total costs due to absenteeism were estimated to be nearly $1.3 million, with an average of $671.4 and a median of $649 The results show that the mean cost of absenteeism was higher among males, patients aged> 50 years, married and physicians. However, the tptal cost of absenteeism was higher among females, patients aged 30–40 years and nurses. This was due to the higher number of women, patients aged 30–40 years and nurses.

**Table 2:** The lost productivity cost ($US) due to absenteeism for the patient with COVID-19 by the sociodemographic variables

Variable	Cost per patient (Mean ± SD)	Median (IQR)	Total cost (% total cost)	Test (***P***-Value)
**Gender**				**T = 3.31** **0.0017**
Male	**688.7 ± 207**	**661.7 (552.5–739.4)**	537,857 (40.9)	
Female	**659.9 ± 193.1**	**625.4 (545.5–728)**	776,729 (59.1)	
**Age**				**F = 98.86** **< 0.0001**
< 30 years	**597.7 ± 136.2**	**594.4 (539–645)**	279,715 (21.3)	
30 to 40 years	**649.1 ± 170.9**	**649.2 (542–708.8)**	629,600 (47.9)	
41 to 50 years	**755.8 ± 208.4**	**737.7 (638.4–851.7)**	313,649 (23.8)	
> 50 years	**872.6 ± 346.2**	**787.8 (616.8–1092)**	91,621 (7)	
**City of residence**				**T = 1.23** **0.2161**
Mashhad	**674.3 ± 209.1**	**658.2 (546.9–733.1)**	1,032,405 (78.5)	
Other cities	**660.8 ± 158.1**	**622.5 (549.6–735.2)**	282,181 (21.5)	
**Workplace**				**F = 1.17** **0.31**
Small hospital (< 200 bed)	**664.1 ± 163**	**630.1 (550.1–740.4)**	444,267 (33.8)	
Large hospital (> 200 bed)	**677.3 ± 230.3**	**631.9 (538.5–742.8)**	757,248 (57.6)	
Medical emergency	**661.2 ± 158.2**	**620.7 (590.8–761.3)**	113,071 (8.6)	
**Marital status**				**F = 10.31** **< 0.0001**
Married	**682 ± 211.5**	**649.1 (543–753.6)**	924,842 (70.4)	
Single	**630.8 ± 190.1**	**611.6 (539.4–662.1)**	247,920 (18.9)	
Other	**678.6 ± 198.7**	**661.7 (655.7–770.2)**	141,824 (10.7)	
**Employment Type**				**T = 18.16** **< 0.0001**
Permanent	**756.2 ± 210.2**	**705.5 (642.9–826.2)**	655,660 (49.9)	
Non-permanent	**604 ± 160.8**	**582.2 (516–661.7)**	658,926 (50.1)	
**Job**				**F = 40.58** **< 0.0001**
Nurse	**693.8 ± 193.1**	**659 (590.3–739.4)**	747,260 (56.8)	
Physician	**827.5 ± 180.5**	**787.8 (651.6–985.1)**	38,894 (3)	
Paramedic staff	**700.7 ± 216.6**	**633.5 (543.9–826.2)**	180,092 (13.7)	
Other staffs	**603.7 ± 183.8**	**560.9 (491.4–661.7)**	348,339 (26.5)	
All patients	**671.4 ± 199.1**	**649 (547.7–733.1)**	1,314,586	–

The regression model results are presented in Table 3. The model results revealed that the variable of gender, age, employment type, job, experience, monthly income, and the number of missed workdays were significant predictors. More than 97% of change by the lost productivity cost of absenteeism due to COVID-19 associated with the included variable. Men faced absenteeism costs on average $3.53 more than women. Employees over the age of 50-year have, on average, about $36 more in lost productivity costs than employees under the age of 30, while these costs were lower for employees aged 30 to 50 years compared to the age group less than 30 years. Moreover, a percent increase in monthly income was associated with an average of $783 increase in the cost of lost productivity. A missed workday resulted in a cost of $37 in the health sector.

**Table 3:** The regression model of determining factors on the lost productivity cost in patients with COVID-19

Variable	Coefficient (CI %95)	*p*-value
**Gender**		
Male	Ref.	
Female	−3.53 (−6.95, −0.11)	0.04
**Age**		
< 30 years	Ref.	
30 to 40 years	−3.17 (−7.59, 1.25)	0.16
41 to 50 years	−2.39 (−8.06, 3.26)	0.4
> 50 years	35.72 (27.71, 43.73)	< 0.0001
**City of residence**		
Mashhad	Ref.	
Other cities	−3.62 (−8.8, 1.56)	0.17
**Workplace**		
Small hospital (< 200 bed)	Ref.	0.44
1Large hospital (> 200 bed)	1.75 (−2.74, 6.26)	0.56
Medical emergency	4.57 (−10.81, 19.96)	
**Marital status**		
Single	Ref.	
Married	−2.98 (−7.1, 1.12)	0.15
Other	−10.13 (−19.64, −0.62)	0.03
**Employment Type**		
Permanent	Ref.	
Non-permanent	14.25 (9.82, 18.67)	< 0.0001
**Job**		
Nurse	Ref.	
Physician	−14 (−24.14, −3.87)	0.007
Paramedic staff	1.77 (−2.82, 6.36)	0.44
Other staffs	16.16 (11.66, 20.67)	< 0.0001
**Experience**		
**< =** 8 year	Ref.	
> 8 year	−8.21 (−12.4, −4.03)	< 0.0001
Not available	−39.01 (−58.19, −19.83)	< 0.0001
Monthly income (Logarithm)	783.11 (771.31–794.91)	< 0.0001
Number of missed work day	37.45 (37.06, 37.84)	< 0.0001
Constant	− 5508.99 (− 5595.81, − 5422.17)	< 0.0001
Number of observations	1958	
R^2^	0.9728	
R^2^ adjusted	0.9726	
F	4086	

## Discussion

To our knowledge, this is the first analysis to provide estimates of the absenteeism costs of COVID-19 among the health care workers in Iran. Our study found 1958 patients with covid-19 between 20 February 2020 and 21 September 2020, with a total of 32,209 days absence of work and an average of 16 days. In this study, the absenteeism cost were estimated $ 1.3 million. Also, the average cost of absenteeism were $671. It is showed that infectious disease could have significant economic impacts through reduced productivity, loss of life, business closures, trade disruption, and decimation of the tourism industry [21]. In previous studies, it is claimed that the COVID-19 can led to increased absenteeism in the workplace. A report on trends in absenteeism during March and April 2020 shown that the COVID-19 causes a significant increase in days absence of work among healthcare personnel and production workers such as meat, poultry, and fish processing workers [22]. In a similar study, Maltezou et al. estimated the cost of absenteeism due to COVID-19 in healthcare personnel in Greece. In this study, a total of 3586 healthcare personnel who exposed to patients with COVID-19 were studied. Among those, there were 254 (6.5%) healthcare personnel with COVID-19. The results of their study showed that of 3332 healthcare personnel, 1332 (40%) were absent from work with a mean duration of 7.5 days. However, among the 254 healthcare personnel with COVID-19, 252 (99.2%) reported absenteeism for a mean duration of 25.8 days. In this study, the cost of absenteeism due to COVID-19 in 254 healthcare personnel were € 552,500 [23]. Gianino et al., conducted a study aimed to estimate the cost of sickness absenteeism during three years of seasonal influenza outbreaks among 16,204 health care workers. The results of this study showed that seasonal influenza can lead to 11,100 days absence of work annually. In this study, the total cost of absenteeism due to seasonal influenza outbreaks among health care workers was € 1.7 million during three years. Also, the average absenteeism cost due to seasonal influenza was € 327 per person [24]. Comparison of the findings of the present study with the above-mentioned study indicated that COVID-19 causes more absenteeism than the flu.

We found that the variable of gender, age, employment type, job, work experience and monthly income had a significant impact on the cost of lost productivity due to absenteeism in the personnel with COVID-19. In a similar study on seasonal influenza, Gianino et al. reported that nurses and allied health professionals and workers in the 40–49 age range were responsible for the most absenteeism costs due to seasonal influenza [24]. Burmeister et al. in their study aimed to determine factors affecting the absenteeism and intent to leave among nurses concluded that less nurse experience and younger age were significant predictors to nurse absenteeism and intent to leave [25]. In another study, it is found that there are a negative association between absenteeism and age [26]. The differences between the findings of the present study and the above studies on the relationship between absenteeism and age may be due to the condition of patients with COVID-19 disease. Elderly people with COVID-19 experience a worse condition than younger patients.

Also, several studies showed that rates of absence are related to socioeconomic status [27]. Kim et al. in a study aimed to determine the absenteeism of financial stress showed that there are a positive and significant relationship between income and absenteeism [27]. This result is consistent with the result of our research. In the study of Kim et al., the variables including age and gender did not relate to absenteeism [27]. A reason for differences in results may be related to differences in the diseases under study. Clark et al. reported that formal and informal education was negatively related to absenteeism [28].

Our study was one of the first studies that estimated the absenteeism cost of COVID-19. One of the strengths of our study was related to the large sample size of study. We studied all personnel of hospitals affiliated to MUMS and selected the personnel that had a sick leave due to COVID-19. However, some limitations should also be noted. The limitation of the study is that the research was conducted in one organization and focused on the cost of absenteeism among the personnel of MUMS hospitals. Due to the nature of research data, the generalizability of findings is limited. Also, some leave cases may not be recorded, which can lead to underestimated absenteeism costs. The distinction between suspected and confirmed cases of COVID-19 was not clear and all cases were considered as COVID-19.

## Conclusion

The results of this study emphasized that COVID-19 can lead to a high economic burden by reducing productivity due to absence from work. Also, there are a positive significant relationship between variables including gender (male), age (> 50 years), employment Type (non-permanent) and monthly income with the absenteeism cost. But, absenteeism cost had a negative relationship with job (physicians) and work experience.

## Data Availability

The datasets used and/or analyzed during the current study are available from the corresponding author on reasonable request.
